# Characterization of *Escherichia coli* RNase H Discrimination of DNA Phosphorothioate Stereoisomers

**DOI:** 10.1089/nat.2021.0055

**Published:** 2021-12-10

**Authors:** Łukasz J. Kiełpiński, Erik Daa Funder, Steffen Schmidt, Peter H. Hagedorn

**Affiliations:** Therapeutic Modalities, Roche Pharma Research and Early Development, Roche Innovation Center Copenhagen, Hørsholm, Denmark.

**Keywords:** RNase H, antisense oligonucleotides, phosphorothioate stereochemistry

## Abstract

Phosphorothioate (PS) modification of antisense oligonucleotides (ASOs) is a critical factor enabling their therapeutic use. Standard chemical synthesis incorporates this group in a stereorandom manner; however, significant effort was made over the years to establish and characterize the impact of chiral control. In this work, we present our in-depth characterization of interactions between *Escherichia coli* RNase H and RNA-DNA heteroduplexes carrying chirally defined PS groups. First, using a massive parallel assay, we showed that at least a single *R*p-PS group is necessary for efficient RNase H-mediated cleavage. We followed by demonstrating that this group needs to be aligned to the phosphate-binding pocket of RNase H, and that chiral status of other PS groups in close proximity to RNase H does not affect cleavage efficiency. We have shown that RNase H's PS chiral preference can be utilized to guide cleavage to a specific chemical bond. Finally, we present a strategy for ASO optimization by mapping preferred RNase H cleavage sites of a non-thioated compound, followed by introduction of *R*p-PS in a strategic position. This results in a cleaner cleavage profile and higher knockdown activity compared with a compound carrying an *S*p-PS at the same location.

## Introduction

RNase H-recruiting antisense oligonucleotides (ASOs) are a promising class of therapeutics, with multiple compounds approved or in development [1]. Current generation of this class of ASOs is heavily modified nucleic acid polymers, typically of length 15–20 nt, designed as “gapmers,” in which a central DNA gap is flanked by nucleotides with unnatural sugar groups, such as locked nucleic acids (LNA), constrained ethyl (cET), or 2′-O-methoxyethyl (MOE) [2]. The DNA gap supports recruitment of RNase H upon hybridization to the target, while the modified flanks are protecting the molecule from exonucleases and increase binding affinity to the target RNA. The oligonucleotide backbone is usually fully modified with phosphorothioate (PS) linkages, in which one of the nonbridging oxygens is replaced by sulfur. This modification provides nuclease resistance and improved cellular uptake and pharmacokinetic properties [3], and is compatible with RNase H activity [4].

Typically, during chemical synthesis of ASOs, sulfur in the PS backbone is incorporated randomly in either *R*p or *S*p configuration (Supplementary Fig. S1), giving rise to a mixture of 2^n−1^ diastereoisomers for an oligonucleotide composed of *n* nucleotides. However, it is possible to synthesize PS oligonucleotides with controlled backbone chirality [5–10]. The specific configuration of the PS group has been associated with distinct biophysical and biochemical properties, such as RNase H activation (*R*p > *S*p), melting temperature (*R*p > *S*p), plasma stability (*S*p > *R*p), immune activation, and *in vitro* and *in vivo* target knockdown activity [5–7,11–13], as well as electrostatic potential as evaluated by quantum-mechanical calculations [14]. On the other hand, nonspecific protein binding seems not to be affected [15]. Recently, Østergaard *et al.* [16] characterized impact of controlling PS chirality on pharmacological and biochemical properties of a CXCL12-targeting gapmer. Despite modulation of the RNase H cleavage patterns, the authors did not see improvements in therapeutically relevant properties.

A crystal structure of the catalytic domain of human RNase H1 complexed with a DNA/RNA duplex reveals that three consecutive phosphate groups of the DNA strand interact directly with the enzyme. The most prominent interaction takes place in the phosphate-binding pocket, which binds the phosphate group located two nucleotides from the scissile phosphate [17] (Supplementary Fig. S2). Nonbridging oxygen atoms of those phosphate groups are prochiral, and, as discussed above, in ASOs, they are modified with chiral sulfur, in either *R*p or *S*p configuration. From interpretation of the enzyme's structure, it has been suggested that a chiral configuration of the three PS groups interacting with the enzyme as 5′-*R*p*S*p*S*p-3′ would promote RNase H1 activity [7].

The tertiary structure of *E. coli* RNase H is remarkably similar to the structure of catalytic domain of human RNase H1 [17], and it has been extensively used as an ersatz of the human enzyme in biochemical assays [18]. In this study, we describe systematic experimental evaluation of *E. coli* RNase H activity in the context of varying ASO PS stereochemistry. We present evidence that similar to what was observed for human RNase H1 [7,16], the *S*p configuration of a PS group docked in the phosphate-binding domain inhibits interaction with RNase H. However, contrary to claims made for human RNase H1, we see that stereoconfiguration of the other two PS groups interacting with the RNase H has negligible effect on the interaction with the enzyme. Our results shed new light on an interplay between base sequence and chiral configuration of ASOs for modulating RNase H activity, and we expect that they will help guide medicinal chemistry efforts for optimizing ASO drug candidates.

## Materials and Methods

For each of the experiments described below, whenever samples were resolved on a gel, it was preceded by the sample undergoing denaturation at 95°C for 2 min and placing on ice. Gel electrophoresis was performed in 1 × TBE buffer on 15% TBE-Urea gel (Novex^®^) with constant 180 V. Bands were visualized using ChemiDoc Touch Imaging System (Bio-Rad) on Blue Sample Tray. The recombinant *E. coli* RNase H enzyme used in the experiments was purchased from Creative BioMart (cat. RNASEH1-433H). It was provided as a solution between 20 and 60 U/μL, but for the concentrations reported below, it was assumed to be 60 U/μL.

### Oligonucleotides used in the study

Sequences and chemical structures of oligonucleotides used in the study are shown on figures or provided in Supplementary Table S1. Stereodefined amidites were obtained by the procedure of Wada and colleagues. [19]. To prepare amidite solutions, stereodefined amidites were dissolved at 0.1 M in 3.5% pyridine in MeCN; std. DNA amidites and std. LNA amidites were dissolved at 0.1 M in MeCN. Synthesis was performed as described previously [20].

The synthesis of the stereodefined oligonucleotides with degenerate positions was performed using a 1:1:1 equimolar mixture of amidites. This resulted in the following 0.1 M amidite solutions: Rp-DNA A, Sp-DNA G, Sp-DNA T (1:1:1), Sp-DNA A, Sp-DNA C, Rp-DNA T (1:1:1), Sp-DNA A, Rp-DNA G, Rp-DNA T (1:1:1), Rp-DNA A, Rp-DNA C, and Sp-DNA T (1:1:1). The solutions were used on the synthesizer using the synthesis protocols described above. Standard commercially available 5′ phosphate modification was used as described by the product manufacturer. Oligonucleotides were purified by standard cartridge purification, followed by standard phosphate deprotection.

### Sequencing method for massive parallel screen of chiral motifs

#### Substrate preparation

A schematic of the procedure for preparation of circular substrates for RNase H is shown in Supplementary Fig. S3A. To prepare a circularized substrate for digestion with RNase H, first the 3p_adapter was ligated to one of four DNA oligonucleotides (L1–L4), by mixing 4.2 μL 100 μM of 3p_adapter with 4.8 μL H_2_O, 3 μL 10 × T4 DNA Ligase Buffer (Thermo Fisher Scientific), 9 μL 50% PEG 4000, 3 μL of 100 μM L1 or L2 or L3 or L4 oligonucleotide, and 6 μL of 100 μL L1_L3_blocker [for reactions with L1 or L3] or L2_L4_blocker [for reactions with L2 or L4]. Samples were incubated as follows: 2 min at 95°C, 5 min at 25°C, and 2 min at 16°C, and placed on ice. To those samples, 20 μL master mix was added (11 volumes of H_2_O, 2 volumes of T4 DNA Ligase Buffer, 6 volumes of 50% PEG 4000, and 1 volume of T4 DNA Ligase HC (Thermo Fisher Scientific)). Samples were incubated as follows: 4 × (2 min at 37°C, 3 min at 30°C, 3 min at 22°C, and 5 min at 16°C), 10 min at 70°C. Fifty microliters 2 × Urea-TBE Loading Dye was added and samples were resolved on a gel for 2 h (Supplementary Fig. S3B). Upper bands (ligated product) were cut from the gel, crushed in 1 × TE buffer, frozen at −80°C for 10 min, and eluted overnight at 4°C (rocking). Samples were downconcentrated on Amicon Ultra 3k columns (Millipore) and nucleic acid concentration was measured with Nanopore 1000 (as dsDNA).

The RNA insert was synthesized by mixing 671 μL H_2_O, 80 μL 10 × reaction buffer (100 μM Tris-HCl pH 8, 20 μM MgCl_2_, 1 M KCl, and 0.02% Tween 20), 1.44 μL 10% Tween 20, 11.8 μL 6.77 μM CS6 GQDSE polymerase [21], 16 μL 10 mM rNTPs (mix of A+C+T for constructs L1 and L3 and mix of A+G+T for constructs L2 and L4), and 20 μL ligated substrate (∼1 μg), and incubating in thermocycler: 1 min at 95°C, 10 min at 68°C, and cooled down to 4°C. Reactions were terminated by addition of 640 μL 50 mM EDTA, concentrated on Amicon Ultra 3k columns, and resolved on a gel for 2 h 45 min (Supplementary Fig. S3C). Bands corresponding to the extended products were extracted and concentrated as ligation products described above.

Next, the 5′ adapter was ligated in a double-ligation reaction, in which the adapter was intended to ligate to both the DNA oligonucleotide 5′ end and synthesized RNA gap 3′ end. Forty microliters of the concentrated, RNA extended product, was mixed with 50 μL of H_2_O and 10 μL 10 μM 5p_adapter_1_3 or 5p_adapter_2_4, for constructs 1 and 3 or 2 and 4, respectively. Samples were heat denatured (5 min at 65°C) and placed on ice. One hundred microliters ligation master mix (7 vol. H_2_O, 8 vol 50% PEG 4000, 4 vol. 10 × T4 DNA Ligase Buffer, and 1 vol. T4 DNA Ligase HC) was added and samples were incubated 4 × (2 min at 37°C, 3 min at 30°C, 3 min at 22°C, and 12 min at 16°C) and placed on ice. Twenty microliters 50 mM EDTA and 80 μL 1 × TE was added and samples were concentrated on Amicon Ultra 3k columns, followed by glycogen-assisted ethanol precipitation. Pellets were dissolved in 10 μL 1 × TE and 10 μL 2 × Urea-TBE loading dye and resolved on gel. Bands corresponding to circularized products were cut out and extracted from the gel as described above, followed by concentrating with Amicon Ultra 3k columns. Circularized products obtained from L1, L2, L3, and L4 will be referred as CS1, CS2, CS3, and CS4, respectively.

#### RNase H hydrolysis and sequencing

RNase H hydrolysis reactions were performed in duplicate. Before hydrolysis, CS1 was pooled with CS4 (mix 1_4), and CS2 was pooled with CS3 (mix 2_3), and the volume as adjusted to 39.6 μL with 1 × TE buffer, to which 4.4 μL 10 × RNH1 buffer (20 mM Tris–HCl pH 7.5, 20 mM KCl, 20 mM 2-mercaptoethanol, 2 mM MgCl_2_, and 0.1 mM EDTA) was added. Samples were split into four 10 μL aliquots. Substrate pools were preheated to 37°C and 10 μL of preheated RNase H master mix (1 volume 10 × RNH1, 1 volume of 2.5 mU RNase H enzyme, and 8 volumes H_2_O) was added. “No enzyme” control was included. Samples were incubated 15 min at 37°C and hydrolysis reaction was terminated by addition of 20.65 μL stop solution (made by mixing 440 μL 2 × Urea-TBE buffer and 7.18 μL 500 mM EDTA). Samples were resolved on gel (4 h), and stained with 1 × SYBR Gold. Upper and lower bands (covalently closed circle and linear form, respectively) were cut, extracted, and concentrated as described above. Two microliters of the samples were used as input for PCR with 19 μL master mix [13.4 volume H_2_O, 2 volume 10 × Standard Taq Reaction Buffer (NEB), 0.4 volume 10 mM dNTP, 1 volume 10 μM RP1 primer, and 0.2 volume Taq DNA polymerase (NEB)] and 1 μL RPIx index primer (indexes 1–16 used). PCR was incubated as follows: 2 min at 95°C, 2 × (30 s at 95°C, 1 min at 50°C, and 1 min at 68°C), 18 × (30 s at 95°C and 30 s at 68°C), and 5 min at 68°C, and hold at 4°C. PCR amplification was confirmed with Bioanalyzer DNA 1000 chip (Agilent). Samples were pooled by adding 15 μL of each PCR sample to 50 μL 50 mM EDTA. The pool was purified with Ampure XP beads (Beckmann Coulter) and sequenced on HiSeq 4000 (Illumina).

#### Data analysis

Sequencing data contained in FASTQ files were demultiplexed requiring perfect match to the index sequence. Adapters were trimmed from the reads with the cutadapt utility [22] requiring adapter match at the 5′ end of the read (“-g ^TATCTGTGCATCC” for CS1 and CS3 or “-g ^TATCTGACGTAGG” for CS2 and CS4) and at the 3′ side (“-a CAGTCAGCTGGAT”). Sequences of length different than nine bases were discarded. Remaining nonamers were required to be composed of only A or G or T nucleotides (CS1 or CS3) or only of A or C or T (CS2 or CS4), and each unique nonamer was counted. Subsequent analysis was performed in the R environment [23]. Positions 1 to 3 and 5 to 7 were extracted and summarized, and reads that have C or G in this stretch were filtered out. Fold changes and false discovery rates of trimer counts were calculated using edgeR [24].

### RNase H-mediated hydrolysis of substrates with eight chiral combinations

**FIG. 2.**
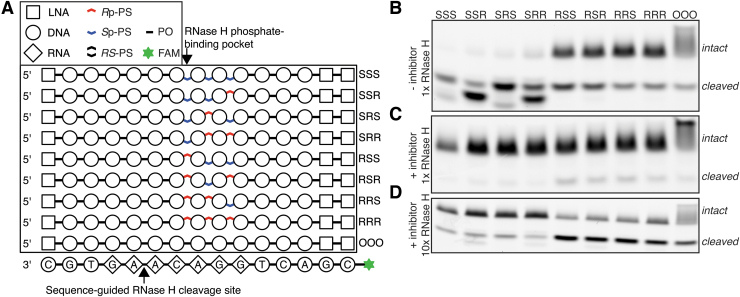
Characterization of RNase H interaction with stereodefined PS backbone. **(A)** Structure of nucleic acid molecules used in the experiment. Base sequence of oligonucleotides in a box is a reverse complement of a sequence of RNA-containing molecule below the box. **(B–D)** Denaturing gel electrophoresis of RNA hydrolysis reactions.

In a total volume of 20 μL, 1 μL of 10 μM HSPA_7RNA oligonucleotide was mixed with 2 μL of 10 μM ASO (chemical structures shown on Fig. 2), 2 μL 10 × annealing buffer, and optionally with 0.2 μL of 100 μM gapO5_der1_nickcirc (competitive inhibitor). Samples were incubated for 2 min at 95°C, then for 10 min at 55°C, then for 10 min at 30°C, and cooled down to 4°C and placed on ice. Next, 20 μL enzyme solution in 1 × RNH1_2 × Mg buffer (containing 50 mU RNase H for 10 × enzyme and 5 mU for 1 × enzyme) was added to the samples, followed by incubation at 30°C for 30 min. Finally, 80 μL urea loading buffer (ULB; 8M Urea, 1 × TBE, and 5 mM EDTA) was added, and samples were resolved on a gel. Exact cleavage sites were assigned by comparison with molecular size marker, prepared by incubating HSPA_7RNA oligonucleotide without the RNase H (in the same reaction buffer as hydrolysis reactions) at 95°C for 60 min, and followed by removal of 2′/3′ phosphate groups [25] with T4 PNK (New England Biolabs).

### RNase H-mediated hydrolysis of substrate with variable position of *R*p linkage

**FIG. 3.**
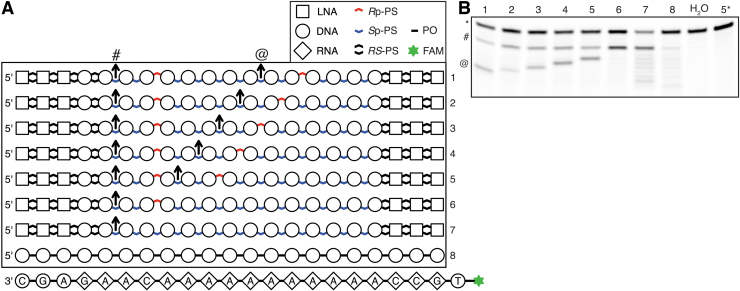
Control of RNase H cleavage site with PS stereoconfiguration. **(A)** Structure of nucleic acid molecules used in the experiment. *Arrows* indicate major RNase H cleavage sites on complementary RNA molecule. **(B)** Denaturing gel electrophoresis of RNA hydrolysis reactions; “*”: full-length product, “#”: internal positive control cleavage site, “@”: cleavage induced by Rp-PS group within a stretch of 11 thymidines. Numbers above lanes indicate used oligonucleotide. Sample “5*” is a “no enzyme” control.

The “chem_walk_RNA” oligonucleotide was cleaved by RNase H in the presence of one of the eight different complementary oligonucleotides (chemical structures shown on Fig. 3). Each reaction was prepared by mixing 1 μL 10 × annealing buffer (200 mM Tris-HCl pH 7.5, 200 mM KCl, 200 mM 2-mercaptoethanol, and 1 mM EDTA), 8 μL H_2_O, 0.5 μL 100 μM chem_walk_RNA oligonucleotide, and 0.5 μL of 1 μM complementary oligonucleotide. Samples were denatured for 2 min at 95°C and placed on ice. Enzyme mix was prepared by mixing 4 volumes of 10 × RNH1_2 × Mg buffer (equal to 10 × annealing buffer supplemented with 40 mM MgCl2), 1 volume of 1 U/μL RNase H, and 35 volumes H_2_O. The “No enzyme” control was treated with an identical mixture, but devoid of the enzyme. Ten microliters of the enzyme mix was added to the tubes with oligonucleotides, and reactions were incubated for 30 min at 30°C, and placed on ice. Forty microliters ULB was added to the reactions and samples were resolved on a gel.

### Sequencing-based detection of RNase H cleavage sites

#### Sample preparation

**FIG. 4.**
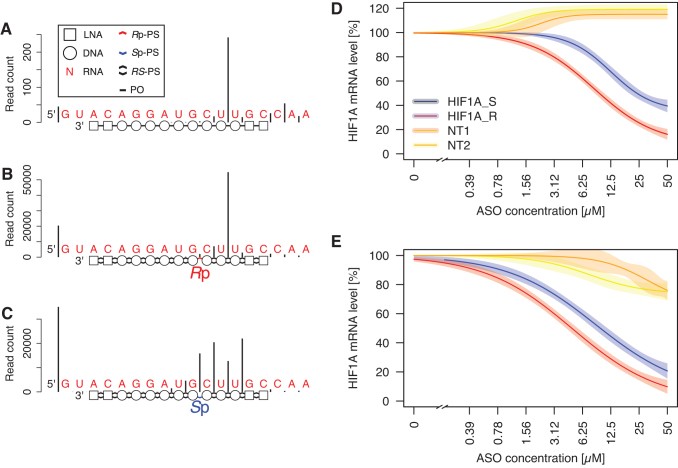
Effects of introducing a single stereodefined PS. Cleavage pattern of RNA molecule complementary to HIF1A ASO as measured with massive parallel sequencing-based method. **(A)** PO compound (HIF1A_O). **(B)** Stereorandom PS with single *R*p-PS (HIF1A_R). **(C)** Stereorandom PS with single *S*p-PS (HIF1A_S). Concentration–response curves of HIF1A mRNA level to ASO treatment in **(D)** A549 cells, and in **(E)** HeLa cells. Shaded areas indicate 95% confidence interval. ASO, antisense oligonucleotide; PO, phosphodiester.

Hydrolysis with RNase H for each sample was performed by mixing 2 μL 10 × annealing buffer, 15 μL H_2_O, 1 μL 10 μM HIF1A_RNA, and 2 μL 10 μM complementary oligonucleotide (chemical structures shown on Fig. 4). Samples were incubated for 2 min at 95°C, for 10 min at 55°C, and for 10 min at 30°C, cooled down to 4°C, and placed on ice. Enzyme mix was prepared by mixing 2 volumes 10 × RNH1_2 × Mg buffer, 17.95 volumes of H_2_O, and 0.05 volume 1 U/μL RNase H. Twenty microliters enzyme mix was added to oligonucleotides, samples were incubated 30 min at 30°C, placed on ice, and reactions were terminated by addition of 10 μL of 25 mM EDTA. Aliquots of the hydrolyzed substrates were mixed with two volumes of ULB and resolved on a gel (Supplementary Fig. S4). Five microliters of the reactions was mixed with 5 μL 10 μM RA5-RNA-rev adapter, incubated 5 min at 95°C, and placed on ice. Ten microliters of ligation master mix [two volumes T4 DNA Ligase buffer, one volume H_2_O, six volumes 50% PEG 4000, and one volume T4 RNA Ligase 2 (MCLAB)] was added and samples were incubated 2 × (37°C for 2 min, 30°C for 3 min, 22°C for 5 min, and 16°C for 10 min), 37°C for 10 min. Reactions were stopped by addition of 5 μL 50 mM EDTA. For reverse transcription, to 2 μL of each ligated sample, mix of oligonucleotides was added (0.5 μL 10 μM revRTP, 1 μL 10 μM HIF1A_decoy, and 6.5 μL H_2_O), samples were denatured 5 min at 95°C, and placed on ice. Five microliters of those samples was transferred to 15 μL RT master mix (4 volumes of 5 × RT buffer, 8.5 volumes H_2_O, 1 volume 100 mM DTT, 1 volume 10 mM dNTP, and 0.5 volume SuperScript III enzyme (Thermo Fisher Scientific)) and incubated 30 min at 42°C followed by 15 min at 50°C and 15 min at 70°C. Two microliters of the RT reaction was transferred to 18 μL PCR (10 volumes 2 × Phusion Polymerase master mix HF (Thermo Fisher Scientific), 2 volumes of 10 μM RP1, 2 volumes of 10 μM respective index primer RPIx, and 4 volumes H_2_O). PCR samples were pooled (with the presence of excess of EDTA), Ampure XP purified, and sequenced on MiniSeq instrument (Illumina). Cautionary note: similar procedure was attempted with a sequence of a chem_walk substrate. However, we have not obtained reads supporting internal cleavage sites. This is likely due to low hybridization affinity of a capture probe to a stretch of oligo-A, which impacted the ligation reaction.

#### Data analysis

Demultiplexed FASTQ files were trimmed with cutadapt (-a TGGAATTCTCGGGTGCCAAGGC-m 14-M 33), filtered with grep to match the fixed parts of the constructs (grep-E “^[ACGT]{6}[AT][CG][GT][AC][AT]GTT”), and lengths of inserts that perfectly match the template were summarized with awk script. Plots were prepared in the R environment.

### HIF1A knockdown in cells

HeLa and A549 cells were plated in 96-well plates 24 h before treatment. The cells were subsequently incubated with ASOs at the indicated concentrations in full cell culture medium. After 72 h, the cells were lysed with 125 μL PureLink Pro lysis buffer and total RNA isolated using the PureLink Pro 96 RNA Kit from Thermo Fisher according to the manufacturer's instructions. Expression of HIF1A mRNA was evaluated in a One Step RT-qPCR using Thermo Fisher TaqMan assay Hs00936368_m1 normalized to expression of GUSB (Hs99999908_m1). All data points were performed in quadruplicates and dose–response curves were fitted with drc R package using the four-parameter log-logistic function fixing HIF1A mRNA level of untreated condition to 100% [23,26].

## Results

### An unbiased screen for RNase H chiral preferences

**FIG. 1.**
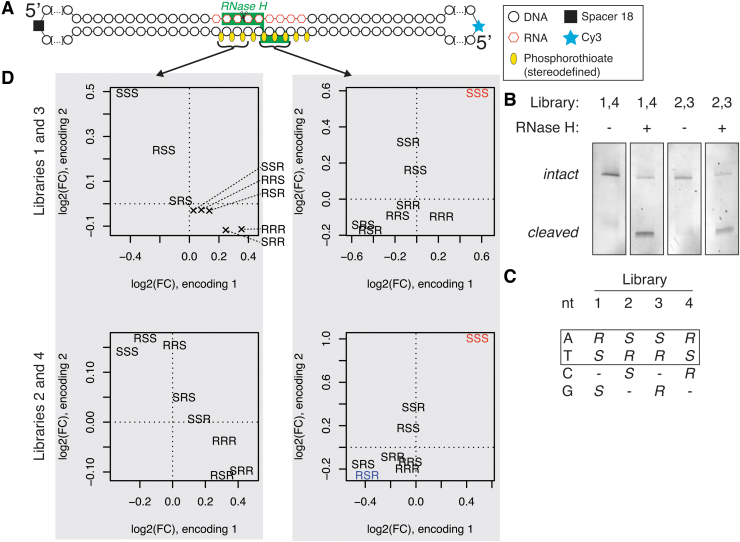
Massive parallel screen for preferred chiral motifs. **(A)** Structure of a circular substrate used in the experiment, with drawing of one of the possible RNase H interaction modes. **(B)** Denaturing gel electrophoresis showing hydrolysis of libraries upon treatment with RNase H. **(C)** Encoding of chiral status by nucleotide identity in different libraries. For the analysis, only the trinucleotides composed exclusively of A or T nucleotides in the randomized region were considered. **(D)** Changes in abundance of chiral motifs at different positions of the randomized gap of the circular substrate upon treatment with RNase H. Encoding 1: adenosine encodes Rp-PS, thymidine encodes Sp-PS; Encoding 2: opposite to encoding 1. Chiral motifs highlighted in *red* have the same effect direction and false discovery rate (FDR) below 0.01 in both of the compared libraries; highlighted in *blue*: as with *red*, but FDR <0.05. PS, phosphorothioate.

We have previously described a method for determining sequence preferences of the *E. coli* RNase H enzyme by hydrolysis and massive parallel sequencing of a pool of all possible sequence combinations of RNA-DNA hybrids, termed H-SPA [27]. For this work, we further developed this method to allow us to study RNase H preferences for PS stereoisomers. We prepared circular nucleic acids, composed of a randomized, 9 nucleotide long, RNA/DNA heteroduplex flanked with fixed DNA sequences (CS1–CS4; Fig. 1A). Within the randomized region, the base identity encodes the stereoconfiguration of the PS group at the 3′ side of the deoxynucleoside. Each position of DNA strand in this region was synthesized as an equimolar mixture of three bases, and each of the four libraries provides different encoding schema (Supplementary Table S1, oligonucleotides L1 to L4). One of the advantages of using a circular substrate is that closed circles have largely distinct electrophoretic mobility in denaturing polyacrylamide gel compared to a linear polymer of the same molecular weight [28].

Two sets of circular substrates were pooled together in a way that allows distinguishing them by sequencing (CS1 mixed with CS4 and CS2 with CS3), and were subjected to limited RNase H hydrolysis (Fig. 1B), followed by gel extraction, PCR amplification, and Illumina sequencing of the constructs. To be able to disentangle a potential interplay between base sequence and PS configuration, we focused on k-mers composed only of A and T nucleotides, and compared substrates for which the only difference is the opposite stereoconfiguration encoding for A and T nucleotide (Fig. 1C). PS stereoconfiguration was deduced from sequenced bases. We calculated changes in abundance of these motifs upon RNase H hydrolysis (Fig. 1D). Comparison of changes in abundance in the region where RNase H interacts with the RNA strand does not reveal any sequence-independent, chirality-dependent effects (Fig. 1D, left panels). Anticorrelation of motifs indicates that in this region, base sequence is a primary driver of cleavage preferences. On the other hand, we see consistent and significant enrichment of stretches of PS in *S*p configuration in the uncleaved fraction of RNase H-treated samples, irrespective of base encoding. These results indicate that presence of at least a single PS linkage in the *R*p configuration in the region where RNase H interacts with phosphate groups is necessary for efficient RNA cleavage in a multiple-substrate competition environment.

### Exhaustive search for optimal chiral configuration

The catalytic domain of human RNase H1 interacts with three consecutive phosphate groups on the DNA strand of RNA-DNA heteroduplex [17]. Based on the understanding of RNase H sequence preferences [27], we were able to design an RNA-DNA substrate, with which RNase H interacts in a specific mode. This allowed us to control the exact position of each of the PS groups with respect to the enzyme. We synthesized oligonucleotides with all eight stereoconfiguration combinations of the three PS groups interacting with the enzyme (Fig. 2A) and tested them in the RNase H cleavage assay. We observed profound differences in cleavage kinetics between substrates having *R*p or *S*p located at the first (from 5′ end on the DNA strand) varied site, which is the site expected to interact with the RNase H phosphate-binding pocket. Specifically, we observed much faster hydrolysis of substrates with *S*p-PS at that position. This result is contrary to the previously proposed 5′-RpSpSp-3′ configuration for optimal RNase H1 activity [7], according to which *R*p-PS should be preferred at this location, and does not agree with our massive parallel screen, indicating that presence of *R*p-PS is critical for efficient cleavage. However, when we repeated the experiment with the presence of a nicked-dumbbell competitive inhibitor of RNase H [27], we saw opposite ranking, with substrates with *R*p-PS in a phosphate-binding pocket being preferentially cleaved (Figs. 2C, D). In both experimental settings, phosphodiester (PO) control behaved similar to compounds with *R*p-PS in the phosphate-binding pocket.

Taken together, these results indicate that *S*p-PS positioned in the phosphate-binding pocket of RNase H leads to weaker association with the enzyme than *R*p-PS or PO at the same site. Similar to our reasoning presented in Supplementary Discussion in the H-SPA article [27], we conclude that a substrate with *R*p-PS in the phosphate-binding pocket behaves as a “preferred” substrate, which exhibits fast association to the intact RNA-DNA duplex (*k*_on_), but slow dissociation (*k*_off_) from the cleaved substrate. On the other hand, substrates with *S*p-PS at this position associate weaker with RNase H (both intact and cleaved), which leads to faster enzyme recycling.

### Configuration of PS linkage guides RNA cleavage site

To assess if it is possible to program RNase H cleavage site using PS stereoconfiguration, we synthesized a series of oligonucleotides with identical base and sugar structures, but with varying PS stereochemistry and used them to cleave complementary RNA oligonucleotides (Fig. 3A). Each of the compounds carries an internal positive control cleavage site (IPCCS), with base sequence optimized for efficient cleavage, and a stretch of 11 deoxythymidines, forming a platform for interactions with RNase H with constant base composition. The majority of the PS groups is in the *S*p configuration, and we placed *R*p-PS groups in various positions across the molecules. For each of the compounds, we observed cleavage of the complementary RNA strand at the IPCCS, including compound 7, in which IPCCS is not augmented by strategically positioned *R*p-PS group (Fig. 3A, B). For compounds 1–5, we see that a cleavage site follows the position of an *R*p-PS group. The cleavage takes place two nucleotides from an *R*p-PS linkage, which again is consistent with a model of *R*p-PS being preferred in the phosphate-binding pocket. Compound 6 has only one *R*p-PS group (two nucleotides from IPCCS), and IPCCS is the only site efficiently cleaved. Compound 7, which did not have any linkage in the *R*p-PS configuration, gave rise to multiple cleavage sites across the RNA molecule. Interestingly, it also led to the most efficient degradation of its RNA substrate. In this experiment, we used 100-fold excess of the RNA substrate over the DNA oligonucleotide, which indicates that the turnover rate, not slowed-down by the tight association of RNase H to *R*p-PS-carrying compounds, was advantageous for promoting efficient RNA hydrolysis.

### Application of PS stereochemistry for rational optimization of ASOs

To apply our understanding from the previous experiments of the RNase H preferences for PS backbone chirality, we set out to optimize a stereorandom LNA-gapmer targeting the HIF1A transcript [29]. First, we determined the RNase H cleavage pattern of an RNA fragment guided by that gapmer's PO version, which gave rise to a single predominant cleavage site (Fig. 4A). Based on our understanding of the importance of the configuration of the PS group in the phosphate-binding pocket for the interaction with RNase H, we synthesized versions of the original compound with a single stereodefined PS group. The compound with a single *R*p-PS group with the remaining PS internucleoside linkages synthesized as a random mixture of R and S configurations using conventional PS phosphoramidite chemistry gave rise to a single predominant cleavage site, akin to the PO version of the compound (Fig. 4B). Oppositely, the compound with a single *S*p-PS group resulted in a drastic change of the cleavage pattern, with a large drop in cleavage rate at the previously observed preferred cleavage site (Fig. 4C).

Next, we assessed the impact of this single modification in a cellular assay for HIF1A knockdown. We established a concentration–response relationship of HIF1A mRNA depletion upon treatment with compounds with a single stereodefined PS bond at a strategic position, two bonds from the predominant *E. coli* RNase H cleavage site (HIF1A_S and HIF1A_R). Both compounds reduced HIF1A expression in two tested cell lines. The estimated concentration needed for achieving 50% knockdown of HIF1A mRNA was two times higher in HeLa cells, and 2.5 times higher in A549 cells for HIF1A_S, compared to a more potent HIF1A_R (Fig. 4D, E).

## Discussion

The PS linkage is a critical modification that enabled development of therapeutic ASOs [3]. Most commonly, it is incorporated in a stereorandom manner. Controlled stereochemistry has been investigated for its impact on pharmacologically relevant properties of ASOs with mixed conclusions [5–7,12,16]; however, there is a consensus in the field that it can be used to expand functional diversity [13,14,16]. In this work, we have focused on the impact of the PS stereoconfiguration on ASO interactions with *E. coli* RNase H, which has a very similar fold to the catalytic domain of human RNase H1 [17], and which has been used extensively in ASO biochemical studies [18].

First, we have shown that in a massive parallel sequencing assay for RNase H hydrolysis, substrates composed exclusively of *S*p-PS linkages in the region where the DNA backbone interacts with RNase H are cleaved significantly less effectively than substrates with at least a single *R*p-PS bond. To unequivocally assign where this critical *R*p-PS linkage is located relative to the cleavage site, we designed an RNA substrate with a base sequence guiding RNase H cleavage toward a specific linkage [27], and synthesized all eight possible combinations of *R*p-PS and *S*p-PS bonds in the three PS bonds interacting with RNase H [17]. At first, our results seemed paradoxical, since in a reaction setting with only the enzyme and RNA-DNA duplex, it was the presence of *S*p-PS linkage at the first position of the motif (in RNase H's phosphate-binding pocket) that led to the most efficient RNA degradation. That is the opposite of what we expected based on the massive parallel sequencing-based experiment and previously published work [7,16]. This inconsistency was reminiscent of our study of RNase H sequence preferences [27], in which base sequence determined to be preferentially cleaved by RNase H in a competition assay resulted in a slow cleavage in isolation. Our interpretation was that a DNA-RNA heteroduplex with a preferred sequence has high affinity to the enzyme before and after the cleavage, which leads to slower enzyme turnover. To check if this was the case also in this setup, we added a competitive inhibitor to the reaction. We assumed that if *R*p-PS in the phosphate-binding pocket leads to increased affinity between substrate and enzyme, it will overcome the competitive inhibition more efficiently than a substrate with *S*p-PS. This is indeed what we have observed, indicating that *S*p-PS in phosphate-binding pocket destabilizes the substrate-enzyme interaction. Interestingly, compared to the major effect of the PS configuration in the phosphate-binding pocket, we see only minor differences in the cleavage extent when modifying the two other PS groups interacting with the enzyme.

It has been previously shown that the 5′-RpSpSp-3′ chiral motif is able to dictate the RNase H cleavage site [16,30]. We set out to demonstrate that the position of the RNA cleavage site can be modulated by the position of the *R*p-PS, irrespective of the base sequence. To show this, we synthesized ASOs with a stretch of 11 identical nucleotides (thymidines) and varying position of *R*p-PS linkage, surrounded by *S*p-PS groups. This approach removes the confounding effect of base sequence in similar experiments [16,30]. For all tested heteroduplexes, the cleavage occurred always two base pairs from the *R*p-PS linkage, further underscoring the importance of the PS stereoconfiguration for RNase H recruitment. We envision that this could be a useful molecular biology tool for induction of RNA cleavages at precisely defined positions. Moreover, when coupled with insights about RNase H sensitivity to base mismatches [31], it may lead to the development of better SNP selective ASOs.

Given the complexity of comparing enzyme kinetics across substrates, it is not straightforward to interpret previously published studies of RNase H activity for substrates of various stereosequences [5–7,16], three of which investigated the activity of human RNase H1. In a study published by Seth and colleagues [6], the authors used large excess of the enzyme; hence the inhibitory effect of slow *k*_off_ should not have influenced the results. However, they modified the entire gap with either all-*R*p-PS or all-*S*p-PS, which impacts not only interactions with the catalytic domain but also with the RNase H1 hybrid-binding domain. In this case, the ranking of cleavage rates induced by three different ASOs was inconsistent across different ASO designs as well as between *E. coli* and human RNase H1. In a study by Verdine and colleagues [7], the authors used only the catalytic domain of human RNase H1 and an excess of substrate, and compared cleavage rates for several diastereomers of a 2′-MOE gapmer. They found that a compound with a repeated 5′-*R*p*S*p*S*p-3′ chiral motif induced hydrolysis of the greatest amount of RNA substrate. In our setup, this chiral motif led to slower cleavage kinetics in the absence of competitive inhibitor. However, dissimilarities between the experimental setups may explain the different results: the base sequence and sugar pattern of ASOs were different, and experiments used similar, although not identical enzymes. Moreover, in our experiment, we have guided RNase H toward a single cleavage site (by optimized base sequence), while Verdine and colleagues did not describe the exact RNase H1 cleavage sites, making it difficult to compare the results. Taken together, we have isolated a single position where PS stereoconfiguration is particularly important for strong interaction with *E. coli* RNase H, while other published studies looked at the end effect of introducing multiple stereoregular PS linkages.

Minor modifications of an ASO chemical structure can significantly influence its pharmacological properties [13]. In this work we provide an attempt of rational optimization of an interaction between RNase H and ASO by controlling the stereoconfiguration of a single, critical PS linkage. First, we experimentally determined RNase H's preferred cleavage site of ASO-RNA duplex. From this position, we inferred which PS group interacts with the enzyme's phosphate-binding pocket, and we synthesized two epimers of the parental ASO with *R*p-PS and *S*p-PS at this location. We found that the RNase H cleavage pattern of complementary RNA was radically different between those two compounds. The *R*p-PS compound preserves native preferred cleavage site, while the *S*p-PS epimer gives rise to alternative cleavage pattern. When tested in a cellular system (which utilizes human RNase H1), we found that the gapmer with *S*p-PS at the location identified to be critical for efficient cleavage by *E. coli* RNase H had the absolute IC_50_ approximately twice as high as the *R*p-PS compound, underscoring the importance of the chiral configuration at that position. This result highlights that it is beneficial to optimize ASO interaction with RNase H to favor strong intermolecular association even at the expense of slowing down dissociation of the complex. It should be noted that the PO version of the gapmer exhibits a single predominant cleavage site, which is not the case for all gapmers, and it will complicate attempts of optimizing RNase H cleavage with stereocontrolled PS backbone. Moreover, any chemical modification introduced in ASO will not only affect the interaction with RNase H but also other pharmacologically relevant properties.

We attempted to understand a structural basis for the preference for *R*p-PS in the phosphate-binding pocket. As presented and illustrated by Verdine and colleagues [7], in the crystal structure of the RNase H1 catalytic domain [17], the prochiral oxygen in a position replaced by sulfur for *R*p-PS compound makes prominent contacts with the side chain of Arg179, which is positively charged at physiological pH. Sulfur at this position will drag the majority of the negative charge of the PS linkage [32] and stabilize the interaction. On the other hand, sulfur in the *S*p-PS position will drag the negative charge away from the oxygen positioned to interact with Arg179, weakening nucleic acid-enzyme interaction. This hypothesis will have to be evaluated with molecular modeling studies.

## Data Availability

Sequencing data are available from European Nucleotide Archive (ENA) at https://www.ebi.ac.uk/ena/browser/view/PRJEB46094.

## Supplementary Material

Supplemental data

Supplemental data

Supplemental data

Supplemental data

Supplemental data
