# Supplementary material for: Characterization of Escherichia coli RNase H Discrimination of DNA Phosphorothioate Stereoisomers
Source: Nucleic Acid Ther. 2021 Dec 10;31(6):383–91. doi: 10.1089/nat.2021.0055 (PMC8713576; doi:10.1089/nat.2021.0055)
Supplement: Supplemental data [file Supp_Fig1.docx]

SUPPLEMENTARY DATA

**Supplementary Figure S1.** Chemical structure of *R*p-PS (red) and *S*p-PS (blue) linkages
