# Supplementary material for: Characterization of Escherichia coli RNase H Discrimination of DNA Phosphorothioate Stereoisomers
Source: Nucleic Acid Ther. 2021 Dec 10;31(6):383–91. doi: 10.1089/nat.2021.0055 (PMC8713576; doi:10.1089/nat.2021.0055)

**Supplementary Figure S2.** Position of human RNase H1 phosphate binding pocket in relation to the scissile phosphate


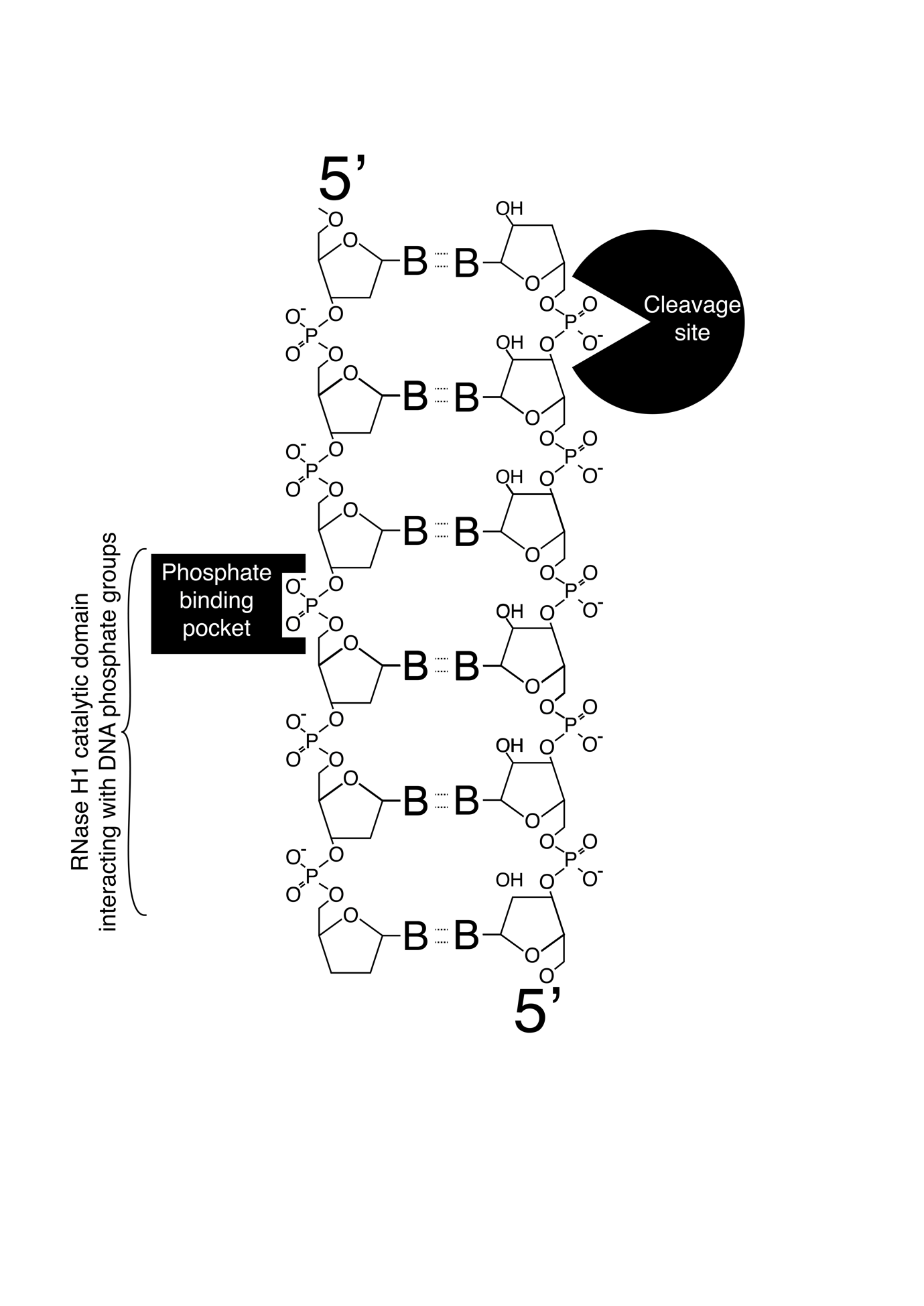

Supplement: Supplemental data [file Supp_Fig2.docx]
