# Supplementary material for: Characterization of Escherichia coli RNase H Discrimination of DNA Phosphorothioate Stereoisomers
Source: Nucleic Acid Ther. 2021 Dec 10;31(6):383–91. doi: 10.1089/nat.2021.0055 (PMC8713576; doi:10.1089/nat.2021.0055)
Supplement: Supplemental data [file Supp_Table1.docx]

**Supplementary Table S1**. Oligonucleotides used in the study. Structure of antisense oligonucleotides (last four rows) is described with an XNAString convention (<https://bioconductor.org/packages/release/bioc/html/XNAString.html>), in which the first row describes the base sequence (E denotes 5meC), second row describes sugar groups (L is LNA, D is DNA), third row is a backbone (O = phosphodiester, X = a non-stereo-controlled phosphorothioate center, S = *S*p phosphorothioate, R = *R*p phosphorothioate)

| **Name** | **Sequence** | **Provider** | **Comments** |
| --- | --- | --- | --- |
| L1 | /5Phos/TGCATCCD*D*D*D*D*D*D*D*D*CAGTCA | in house | D = 1:1:1 of Rp-DNA A, Sp-DNA G, Sp-DNA T |
| L2 | /5Phos/ACGTAGGH*H*H*H*H*H*H*H*H*CAGTCA | in house | H = 1:1:1 of Sp-DNA A, Sp-DNA C, Rp-DNA T |
| L3 | /5Phos/TGCATCCD*D*D*D*D*D*D*D*D*CAGTCA | in house | D = 1:1:1 of Sp-DNA A, Rp-DNA G, Rp-DNA T |
| L4 | /5Phos/ACGTAGGH*H*H*H*H*H*H*H*H*CAGTCA | in house | H = 1:1:1 of Rp-DNA A, Rp-DNA C and Sp-DNA T |
| 3p_adapter | /5Phos/GCTGGATTGGAATTCTCGGGTGCCAAGG/iCy3/ GTTCAGAGTTCTACAGTCCGACGATCATCCAGCTGACTG | IDT DNA |  |
| L2_L4_blocker | DDDDDDDDDCCTACGT/3AmMO/ | IDT DNA | D = A or G or T |
| L1_L3_blocker | HHHHHHHHHGGATGCA/3AmMO/ | IDT DNA | H = A or C or T |
| 5p_adapter_1_3 | /5Phos/GGATGCACAGATACACCTGACACCCGTGGA/iSp18/ GTTCAGAGTTCTACAGTCCGACGATCTATCTG | IDT DNA |  |
| 5p_adapter_2_4 | /5Phos/CCTACGTCAGATACACCTGACACCCGTGGA/iSp18/ GTTCAGAGTTCTACAGTCCGACGATCTATCTG | IDT DNA |  |
| RP1 | AATGATACGGCGACCACCGAGATCTACACGTTCAGAGTT CTACAGTCCGA | IDT DNA |  |
| RPIx | CAAGCAGAAGACGGCATACGAGATxxxxxxGTGACTGGAG TTCCTTGGCACCCGAGAATTCCA | IDT DNA | xxxxxx is illumina index sequence |
| gapO5_der1_nickcirc | /5Phos/rArGmGGTAGCTCACGCTAGTGAGCTACCCTTGTT CCCCGACGTGCGCTAGCACGTCmGmGmGmGmAmArCrA | IDT DNA |  |
| HIF1A_RNA | /5Phos/rGrUrArCrArGrGrArUrGrCrUrUrGrCrCrArAACWKMSW NNNNNNGATCGTCGGACTGTAGAAC/36-FAM/ | IDT DNA |  |
| HIF1A_decoy | TGGCAAGCATCCTGTA | IDT DNA |  |
| revRTP | GTTCAGAGTTCTACAGTCCGACGATC | IDT DNA |  |
| RA5-RNA-rev | NNNNNNTGGAATTC/iSp18/CCTTGGCACCCGAGAATrUrCrCrA | IDT DNA |  |
| chem_walk_RNA | /56-FAM/TrGrCrCrArArArArArArArArArArArCrArArGAGC | IDT DNA |  |
| HSPA_7RNA | /56-FAM/CGACTrGrGrArCrArArGTGC | IDT DNA |  |
| HIF1A_O | GEAAGCATCCTGT  LLDDDDDDDDDLL  OOOOOOOOOOOO | in house |  |
| HIF1A_X | GEAAGCATCCTGT  LLDDDDDDDDDLL  XXXXXXXXXXXX | in house |  |
| HIF1A_R | GEAAGCATCCTGT  LLDDDDDDDDDLL  XXXXRXXXXXXX | in house |  |
| HIF1A_S | GEAAGCATCCTGT  LLDDDDDDDDDLL  XXXXSXXXXXXX | in house |  |
| NT1 | TTGAATAAGTGGATGT  LLLDDDDDDDDDDLLL  XXXXXXXXXXXXXXX | in house | Non-targeting control ASO |
| NT2 | TGATAAGACATTTATT  LLLLDDDDDDDDLLLL  XXXXXXXXXXXXXXX | in house | Non-targeting control ASO |

| **Key:** |  |  |  |
| --- | --- | --- | --- |
| * = PS linkage | /36-FAM/ = 3' FAM | /iSp18/ = internal spacer 18 | mN = 2'-O-Methyl |
| /5Phos/ = 5' phosphate | /3AmMO/ = 3' amino | /56-FAM/ = 5' FAM | rN = ribonucleotide |
