# Supplementary material for: Characterization of Escherichia coli RNase H Discrimination of DNA Phosphorothioate Stereoisomers
Source: Nucleic Acid Ther. 2021 Dec 10;31(6):383–91. doi: 10.1089/nat.2021.0055 (PMC8713576; doi:10.1089/nat.2021.0055)

**Supplementary Figure S3.** Preparation of circular substrate of RNase H. (A) To prepare an RNase H substrate, we: (1) ligated 3’ adapter, (2) synthesized RNA gap complementary to the randomized DNA region using an enzyme with primer-dependent, DNA-templated RNA polymerase activity and a mixture of three ribonucleotides. This led to synthesis termination upon reaching the end of the randomized region, where fourth ribonucleotide was necessary to continue RNA strand synthesis; (3) 5’ adapter double-ligation, which produced covalently closed circle. (B) Gel electrophoresis of ligation of the 3’ adapter. (C) Gel electrophoresis of RNA gap synthesis. (D) Gel electrophoresis of double ligation of 5’ adapter. Only the top band was cut out of the gel and extracted. (C and D) Lanes marked as ‘-‘ are unprocessed inputs to the analyzed reactions. Loading amounts were not normalized. For panel C “unligated 3p_adapter” band was present but is not shown.


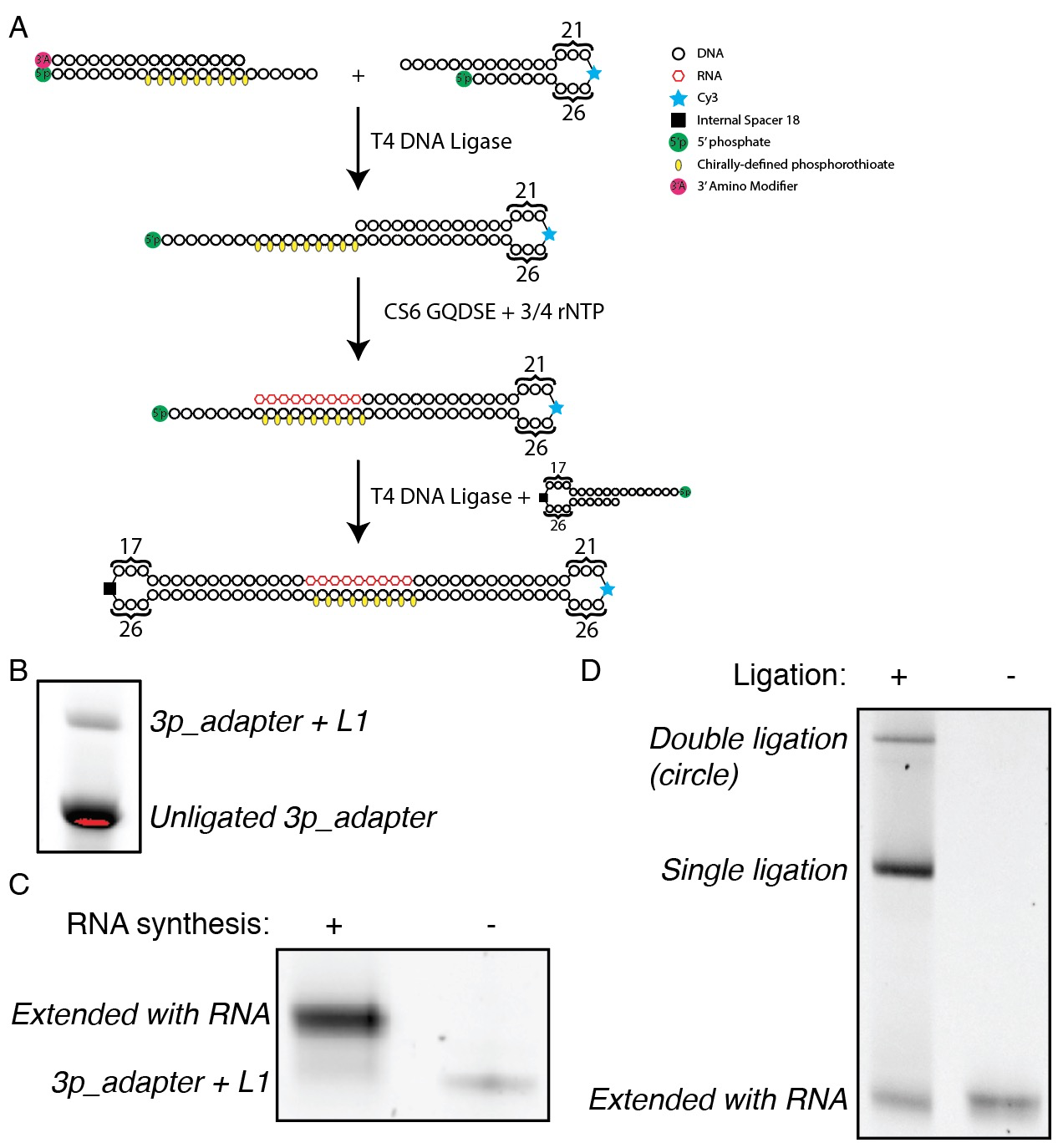

Supplement: Supplemental data [file Supp_Fig3.docx]
