# Supplementary material for: Characterization of Escherichia coli RNase H Discrimination of DNA Phosphorothioate Stereoisomers
Source: Nucleic Acid Ther. 2021 Dec 10;31(6):383–91. doi: 10.1089/nat.2021.0055 (PMC8713576; doi:10.1089/nat.2021.0055)

**Supplementary Figure S4.** Gel electrophoresis of RNase H-digested HIF1A mRNA fragment substrate prior to sequencing library preparation.


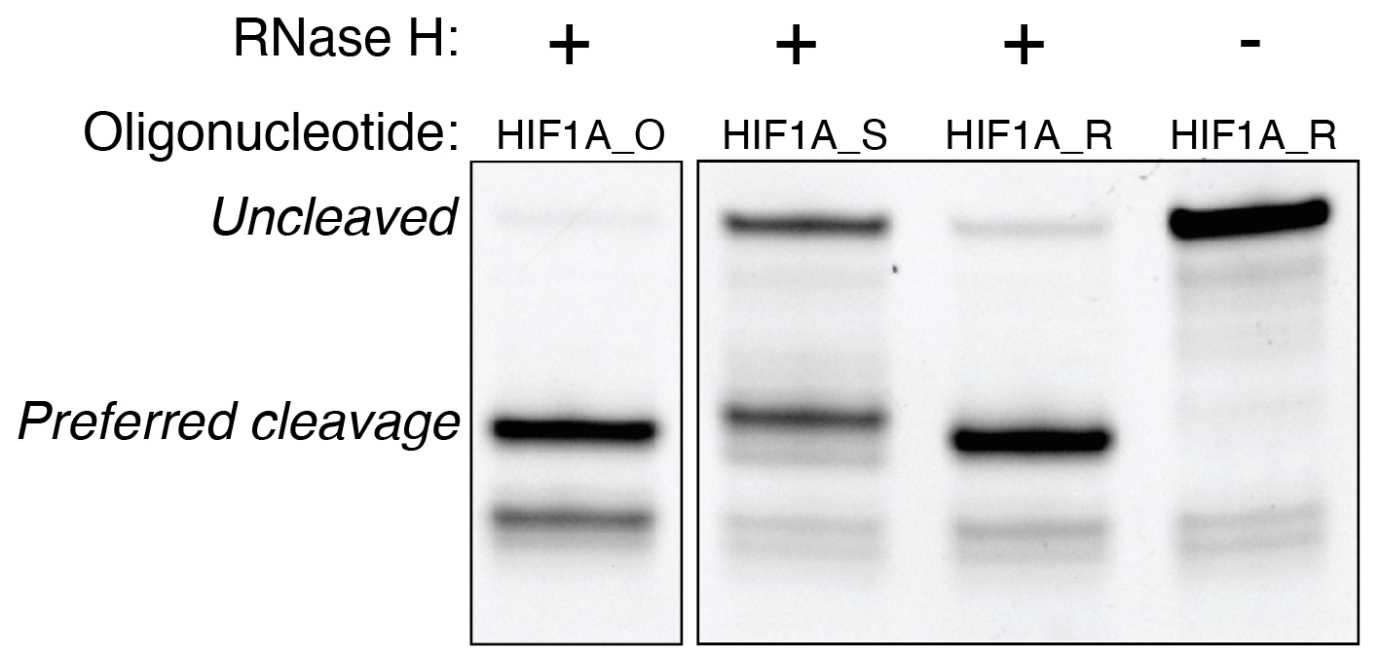

Supplement: Supplemental data [file Supp_Fig4.docx]
